# Effect of Pregelatinized Rice Starch on the Quality and Freeze–Thaw Stability of Glutinous Rice Dumplings

**DOI:** 10.3390/foods15213929

**Published:** 2026-11-02

**Authors:** La Luo, Yizheng Zhou, Zhiyi Zhou, Yufeng Fu, Mengyao Hao, Jiali Xing, Li Wang, Kai Huang, Xiaohu Luo, Dan Ouyang

**Affiliations:** 1 Zhejiang-Malaysia Joint Research Laboratory for Agricultural Product Processing and Nutrition, College of Food Science and Engineering, Ningbo University, Ningbo 315832, China; 18758387919@163.com (L.L.); 246004665@nbu.edu.cn (Y.Z.); 246003958@nbu.edu.cn (Z.Z.); 246000678@nbu.edu.cn (Y.F.); 246000675@nbu.edu.cn (M.H.); xh06326@jiangnan.edu.cn (X.L.); 2 Ningbo Key Laboratory of Detection, Control, and Early Warning of Key Hazardous Materials in Food, Ningbo Academy of Product and Food Quality Inspection (Ningbo Fibre Inspection Institute), Ningbo 315048, China; hellojiali77@gmail.com; 3 School of Food Science and Technology, Jiangnan University, 1800 Lihu Avenue, Wuxi 214122, China; wangli@jiangnan.edu.cn; 4 School of Health Science and Engineering, University of Shanghai for Science and Technology, Shanghai 200093, China; hkjn1990@163.com

**Keywords:** pregelatinized starch, freeze–thaw stability, glutinous rice dumplings, water distribution, microstructure, textural properties

## Abstract

The rapid expansion of the frozen glutinous rice dumpling (GRD) industry faces the challenge of quality deterioration caused by freeze–thaw cycles during product distribution. Pregelatinized starch has been reported to improve the freeze–thaw stability of starchy foods, but its effect on GRDs remains unclear. In this study, rice starch, which is widely available and low in cost, was used to prepare pregelatinized rice starch (PGRS) to enhance the quality and freeze–thaw stability of GRDs. The effect of adding 2–8% PGRS on GRD quality over 1, 3, and 5 freeze–thaw cycles was investigated using texture analysis, ultraviolet spectrophotometry, differential scanning calorimetry, rheometry, Fourier transform infrared spectroscopy, low-field nuclear magnetic resonance, and scanning electron microscopy. Results showed that appropriate PGRS addition weakened an ordered gel structure, increased bound water content, improved microstructural stability, and reduced gelatinization enthalpy. Throughout testing, PGRS reduced cracking and water loss, enhanced textural properties, and increased soup transparency. This confirms that PGRS can serve as a functional ingredient for improving GRD quality and freeze–thaw stability, offering valuable insights for industrial production.

## 1. Introduction

Glutinous rice dumplings (GRDs), a traditional Chinese delicacy, are widely favored for their soft and sticky texture, which is attributed to the high amylopectin content (up to 98%) of glutinous rice endosperm starch [1]. With the development of the frozen food industry, quick-freezing technology has been widely adopted to extend the shelf life of GRDs and meet consumer demand for convenience. However, during actual storage, transportation, and distribution, frozen GRDs inevitably experience temperature fluctuations, leading to repeated freeze–thaw cycles (FTCs), a primary challenge that causes progressive quality deterioration. After multiple FTCs, GRDs exhibit pronounced defects, including surface micro-cracks, filling leakage, increased brittleness of raw dumplings, and hardness and loss of elasticity after cooking, as well as thick and turbid soup [2]. Such quality deterioration not only reduces consumer acceptance but also results in significant economic losses.

Currently, methods to improve GRD quality mainly involve optimizing the freezing process [3], incorporating food additives [4], and improving production techniques [5]. Physical treatments, including electron beam irradiation and dielectric barrier discharge cold plasma treatment, have also been investigated for improving rice eating quality and modifying starch properties [6,7]. Among these, the addition of food additives (such as modified starches, hydrocolloids, emulsifiers, and enzymes) is the most practical and widely adopted approach [8,9]. Nevertheless, despite their effectiveness, the growing consumer preference for “clean-label” products has raised concerns over potential chemical residues from synthetic additives, driving the search for natural, minimally processed, and green alternatives.

Pregelatinized starch, a physically modified starch obtained via moist-heat treatment, exhibits enhanced water-holding capacity and retrogradation resistance, making it a promising natural and clean-label quality improver [10,11,12]. Several studies have supported its application in frozen foods. Wang et al. [12] demonstrated that pregelatinized starch improves gel network formation, reducing cracking and water loss in GRDs, while Li et al. [13] showed that it lowers freezable water content and inhibits ice crystal growth in frozen dough, thereby enhancing texture after frozen storage. Among various starch sources, rice starch is particularly attractive due to its wide availability, low cost, good biocompatibility, and inherent freeze–thaw stability [14,15,16,17]. Therefore, the combination of rice starch with pregelatinization offers a potential strategy. Ding et al. [18] showed that pregelatinizing part of the rice flour within an otherwise unchanged formulation modified batter structure and improved rice bread quality, supporting the functional contribution of pregelatinization itself. Heat treatment can also modify interactions between rice starch and protein, along with the resulting gel properties [19]. However, systematic research on the effects of pregelatinized rice starch (PGRS) on the freeze–thaw stability of GRDs, specifically under repeated FTCs, remains lacking.

Therefore, in this study, PGRS was prepared from rice starch using a green physical pregelatinization technology and incorporated into frozen GRDs. Focusing on repeated FTCs as a specific stress condition, we systematically evaluated the effects of different PGRS substitution levels on GRD quality, including cracking rate, cooking properties, texture, and water distribution, relative to the formulation without PGRS, and examined the associated changes in starch structure and water mobility. The findings are expected to provide a theoretical basis for developing a green and efficient modifier for use in frozen GRDs to mitigate the detrimental effects of repeated FTCs, with significant implications for advancing the quality of frozen rice and flour products.

## 2. Materials and Methods

### 2.1. Materials

The materials used in our work comprise rice starch (Shanghai Yuanye Biotechnology Co., Ltd., Shanghai, China), deionized water, glutinous rice flour (Ningbo Gangyagou Food Co., Ltd., Ningbo, China), and potassium bromide (KBr, purity > 99%, Aladdin Biochemical Technology Co., Ltd., Shanghai, China).

### 2.2. Instruments

The instruments used in this work were as follows: a texture analyzer (TA.XT Plus, Stable Micro Systems, Godalming, UK), an ultraviolet–visible (UV–Vis) spectrophotometer (LAMBDA850+, PerkinElmer, Shelton, CT, USA), a differential scanning calorimeter (DSC3500, NETZSCH, Selb, Germany), a Fourier transform infrared (FT-IR) spectrometer (Nicolet iS20, Thermo Fisher, Waltham, MA, USA), a low-field nuclear magnetic resonance (LF-NMR) analyzer (NMI200-060H-1, Niumai Analytical Instrument Co., Ltd., Suzhou, China), a rheometer (DHR-2, TA Instruments, New Castle, DE, USA), a freeze dryer (Christ ALPHA 1-4 LDplus, Martin Christ, Osterode am Harz, Germany), an electric thermostatic water bath (DK-S22, Jinghong Experimental Equipment Co., Ltd., Shanghai, China) and a scanning electron microscope (SEM, Hitachi TM4000 IIplus, Hitachi, Co., Ltd., Tokyo, Japan).

### 2.3. Methods

#### 2.3.1. Preparation of PGRS

The preparation of PGRS was carried out according to the method of Yulianingsih and Gohtani [20], with modifications. Rice starch was mixed thoroughly with water to prepare a 5% (*w*/*w*) suspension. The suspension was then heated in a water bath at 85 °C for 10 min under continuous stirring to ensure uniform heat transfer, allowing the gelatinization process to proceed fully and evenly. Subsequently, the mixture was rapidly cooled in ice water for 3 min. The obtained mixture was poured onto a stainless steel tray and dried in an oven at 40 °C for 12 h. The dried product was ground and passed through a 100-mesh sieve to obtain the final sample.

#### 2.3.2. Preparation of GRDs

Based on the method of Wang et al. [12], with slight modifications, fresh GRDs were prepared. The PGRS obtained in Section 2.3.1 was substituted for glutinous rice flour (dry basis) at ratios of 0%, 2%, 4%, 6%, and 8% and was mixed with deionized water (wet basis) at a dry-to-wet mass ratio of 5:4. The mixture was uniformly blended and kneaded to form a fresh rice dough (200 g). Portions of dough weighing 10 g were hand-rolled into round shapes to produce 20 GRDs. The obtained GRDs exhibited a natural color, with a smooth and crack-free surface, uniform size, and intact structure. The sample with 0% addition served as the control group, while those with 2%, 4%, 6%, and 8% additions constituted the experimental groups.

#### 2.3.3. The Freeze–Thaw Cycle of GRD

The prepared GRDs were frozen at −20 °C for 21 h and then thawed in an incubator at 25 °C for 3 h to complete one FTC. This cycle was repeated 0, 1, 3, and 5 times, labeled as FTC-0, FTC-1, FTC-3, and FTC-5, respectively [21].

#### 2.3.4. Native Rice Starch Control

A native rice starch control was prepared by replacing 6% of the glutinous rice flour with native rice starch on a dry basis. Dumpling preparation and freeze–thaw treatments followed the procedures described in Section 2.3.2 and Section 2.3.3, respectively, with the water addition and processing conditions kept consistent with those of the PGRS treatments. Textural properties, cracking rate, and water loss rate were determined according to the texture profile analysis (TPA), cracking rate, and water loss rate.

The results were compared with the data for the control and the 6% PGRS treatment under the corresponding FTC conditions.

#### 2.3.5. Quality Parameters of GRDs

##### Texture Profile Analysis (TPA)

Five frozen GRD were boiled in 500 g of deionized water (water-to-GRD ratio of 10:1, *w*/*w*) for 4 min. After cooking, the GRDs were removed and cooled to room temperature prior to measurement. Texture profile analysis (TPA) of cooked GRDs was conducted using a compression mode with a P/36R cylindrical probe (30 mm height). The pre-test, test, and post-test speeds were set to 2.0 mm/s, 1.0 mm/s, and 1.0 mm/s, respectively. A trigger force of 5 g was applied. The samples were compressed to 50% of their original height, with a 5 s interval between the two compression cycles.

##### Transmittance of the Soup After Boiling

The soup obtained from cooking the GRDs was cooled to room temperature and made up to the mark in a 500 mL volumetric flask. The transmittance (%) was then measured at 650 nm using an ultraviolet spectrophotometer, with distilled water as the reference.

##### Cracking Rate and Water Loss Rate

After completion of the designated freeze–thaw treatment, ten GRDs from each PGRS substitution group (0%, 2%, 4%, 6%, and 8%; *N* = 10 per group) were visually inspected. Each GRD was assigned an integrity score according to its surface condition: 1.0 for an intact sample without visible cracking, 0.5 for a partially cracked sample showing an intermediate degree of damage, and 0 for a completely ruptured sample. The weighted cracking rate was calculated as follows:

(1) $$Cracking\;rate\;\left(\%\right)=\left(N_{0}+0.5N_{0.5}\right)/N\times 100,$$

where *N*_0_ is the number of completely ruptured GRDs (integrity score 0), *N*_0.5_ is the number of partially cracked GRDs (integrity score 0.5), and *N* is the total number of GRDs inspected. Intact GRDs (integrity score 1.0) contribute zero to the numerator.

Water loss during freeze–thaw treatment was evaluated from the difference in sample mass before and after processing. Samples were weighed before initial freezing and at the corresponding frozen endpoints, with care taken to minimize adhesion and material loss during handling. The water loss rate was calculated as follows:

(2) $$A\left(\%\right)=\left(X-X_{1}\right)/X\times 100,$$

where *A* is the water loss rate (%), *X* is the sample mass before initial freezing, and *X*_1_ is the sample mass at the corresponding frozen endpoint. For FTC-0, *X*_1_ was measured after initial freezing and before any thawing. The values for FTC-1, FTC-3, and FTC-5 represent cumulative water loss over the corresponding treatment, including final refreezing.

##### Differential Scanning Calorimetry (DSC)

A 3 mg portion of powder obtained by grinding freeze-dried GRDs was weighed into a DSC aluminum pan, and 12 μL of deionized water was added. After sealing, the sample was allowed to equilibrate at 25 °C for 24 h. An empty pan was used as the reference, and the sample pan was heated from 30 °C to 100 °C at a scanning rate of 10 °C/min. The thermogram was analyzed using the software supplied with the DSC instrument to determine the enthalpy change (ΔH), onset temperature (To), peak temperature (Tp), and end temperature (Te).

##### Dynamic Rheological Property Measurement

The freeze-dried powder was prepared into a 12% concentration composite emulsion and heated for gelatinization. A parallel plate system, 40 mm in diameter, was used, with a gap of 1.0 mm and strain set at 2%. Frequency sweep (0.1~10 Hz) was conducted at 25 °C to determine the storage modulus (G′) and loss modulus (G″) of the samples. The ratio of G″/G′ represents the loss tangent value (tan δ). Each treatment was measured in triplicate (*n* = 3).

#### 2.3.6. Mechanism Investigation

##### Fourier Transform Infrared Spectroscopy (FT-IR)

The structural information of freeze–thawed GRDs was analyzed using an FT-IR spectrometer. The freeze-dried powder was mixed with KBr at a 1:100 (*w*/*w*) ratio. Samples were scanned in the range of 400–4000 cm^−1^ via the pressing plate method, with transmittance recorded at 1047 cm^−1^ and 1022 cm^−1^ [22].

##### Low-Field Nuclear Magnetic Resonance (LF-NMR)

The water distribution in the GRDs was determined using LF-NMR. The raw GRD samples were placed in glass tubes, and the spin–spin relaxation time (T_2_) was acquired using the Carr–Purcell–Meiboom–Gill (CPMG) sequence. The parameter settings were as follows: the main frequency (SF) = 21 MHz, P1 = 15 μs (90°pulse width), P2 = 25.04 μs (180°pulse width), receiver bandwidth (SW) = 200 kHz, and echo time (TE) = 0.2 ms [23].

##### Scanning Electron Microscopy (SEM)

The freeze-dried powder were evenly dispersed on double-sided adhesive tape mounted on a copper stub and subsequently sputter-coated with a thin layer of gold–palladium. The micrographs were observed at 5.0 kV accelerating voltage and 1000× magnification.

#### 2.3.7. Statistical Analysis

**Figure 1:**
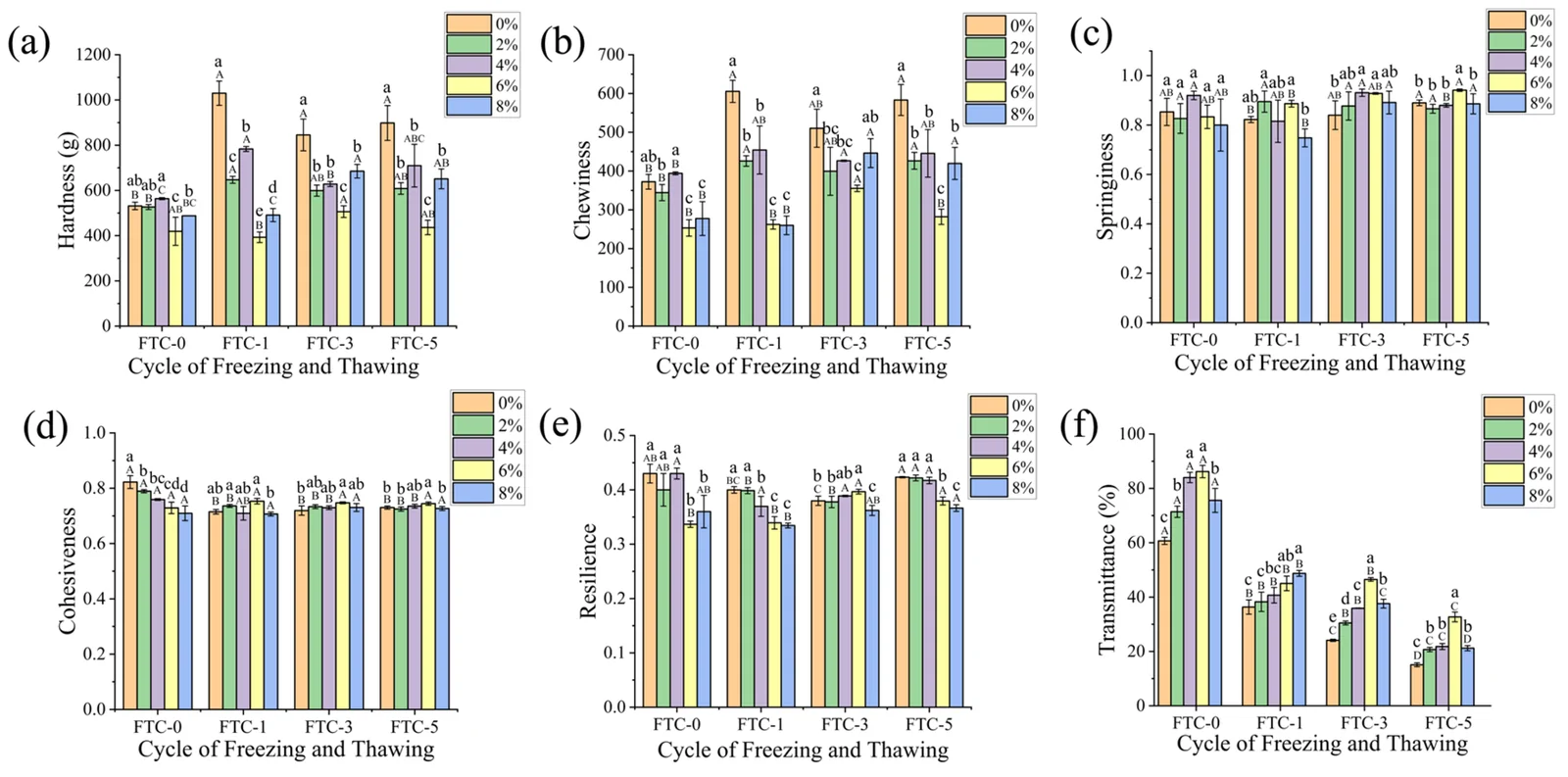
Effects of PGRS substitution on (**a**) hardness, (**b**) chewiness, (**c**) springiness, (**d**) cohesiveness, (**e**) resilience, and (**f**) soup transmittance of glutinous rice dumplings subjected to different numbers of freeze–thaw cycles (FTCs). Data are presented as means ± standard deviations (SD), with three replicate measurements per treatment (*n* = 3). Different lowercase letters indicate significant differences among PGRS substitution levels within the same FTC level, determined by one-way ANOVA followed by Duncan’s multiple range test. Different uppercase letters indicate significant differences among FTC levels within the same PGRS substitution level, determined by Welch’s one-way ANOVA followed by Games–Howell multiple comparisons (*p* < 0.05). Groups sharing at least one letter within the corresponding comparison are not significantly different.

For Figure 1, comparisons among PGRS substitution levels within the same FTC level were performed using one-way analysis of variance (ANOVA) followed by Duncan’s multiple range test. Comparisons among FTC levels within the same PGRS substitution level were performed using Welch’s one-way ANOVA followed by Games–Howell multiple comparisons. For the three treatment groups in Table 1, Welch’s one-way ANOVA followed by Games–Howell multiple comparisons, was used; the sample sizes were *n* = 3 for the control, *n* = 6 for native rice starch, and *n* = 3 for PGRS. Statistical significance was set at *p* < 0.05 for these comparisons.

## 3. Results and Discussion

### 3.1. Texture Profile Analysis

The textural properties of GRDs serve as critical indicators for evaluating their eating quality after cooking [24]. As shown in Figure 1a,b, the hardness and chewiness of GRD generally decreased as the PGRS substitution ratio increased from 0% to 6%, followed by an increase at 8%. The 6% PGRS group exhibited relatively low hardness and chewiness across all freeze–thaw conditions, indicating that moderate PGRS substitution was effective in reducing the firmness and chewing resistance of GRDs. This phenomenon can be attributed to the formation of a gel network by PGRS within the dough matrix, which reduced structural rigidity while concurrently improving softness and extensibility [25]. Furthermore, the water-absorbing capacity of PGRS effectively suppressed ice crystal growth and recrystallization, mitigating texture deterioration induced by ice crystal damage to the starch matrix during freeze–thaw cycles, thereby decelerating the escalation of hardness and chewiness.

Springiness is the ability of a sample to return to its original state after being compressed [26]. Under certain freeze–thaw conditions, springiness and cohesiveness differed among groups with different PGRS substitution ratios, but neither parameter exhibited a consistent trend across all freeze–thaw conditions. Resilience showed a general declining trend with increasing PGRS content (Figure 1e). This decline may stem from the introduction of amorphous regions by PGRS, which disrupted the native crystalline structure of glutinous rice starch. Such structural perturbation compromised the homogeneity of the starch gel network and impaired molecular alignment, ultimately reducing elastic recovery capacity [12].

### 3.2. Transmittance of the Soup After Boiling

Soup transmittance serves as an indicator of the diffusion and dissolution of surface glutinous rice flour into the cooking water [27]. The weak hydrogen bonds in the crystalline micellar area were destroyed by heating during the cooking process. The starch granules were ruptured after hydration and swelling by water absorption; thus, most of the amylose leached from the starch granule to the soup, decreasing the light transmittance. As shown in Figure 1f, soup transmittance generally decreased with increasing freeze–thaw cycles. At the same number of freeze–thaw cycles, the addition of PGRS generally increased soup transmittance, although the extent of improvement depended on the substitution ratio. At FTC-1, transmittance increased with increasing PGRS substitution and reached its maximum at 8%. At FTC-3 and FTC-5, the 6% PGRS group exhibited the highest transmittance, followed by a decrease at 8%. Compared with the control group, all PGRS-containing groups exhibited significantly higher transmittance at FTC-3 and FTC-5. Moreover, the more freeze–thaw cycles were conducted, the more pronounced this difference became. This fully confirms that PGRS has a stable effect on improving the soup clarity of GRDs after freeze–thaw treatment. This improvement likely stems from PGRS’s high water-absorption and water-retention capacities, which promote tighter water binding within the dumplings. Consequently, this reduces surface cracking and mitigates starch leaching, thereby enhancing optical clarity [12].

### 3.3. Cracking Rate and Water Loss Rate

**Figure 2:**
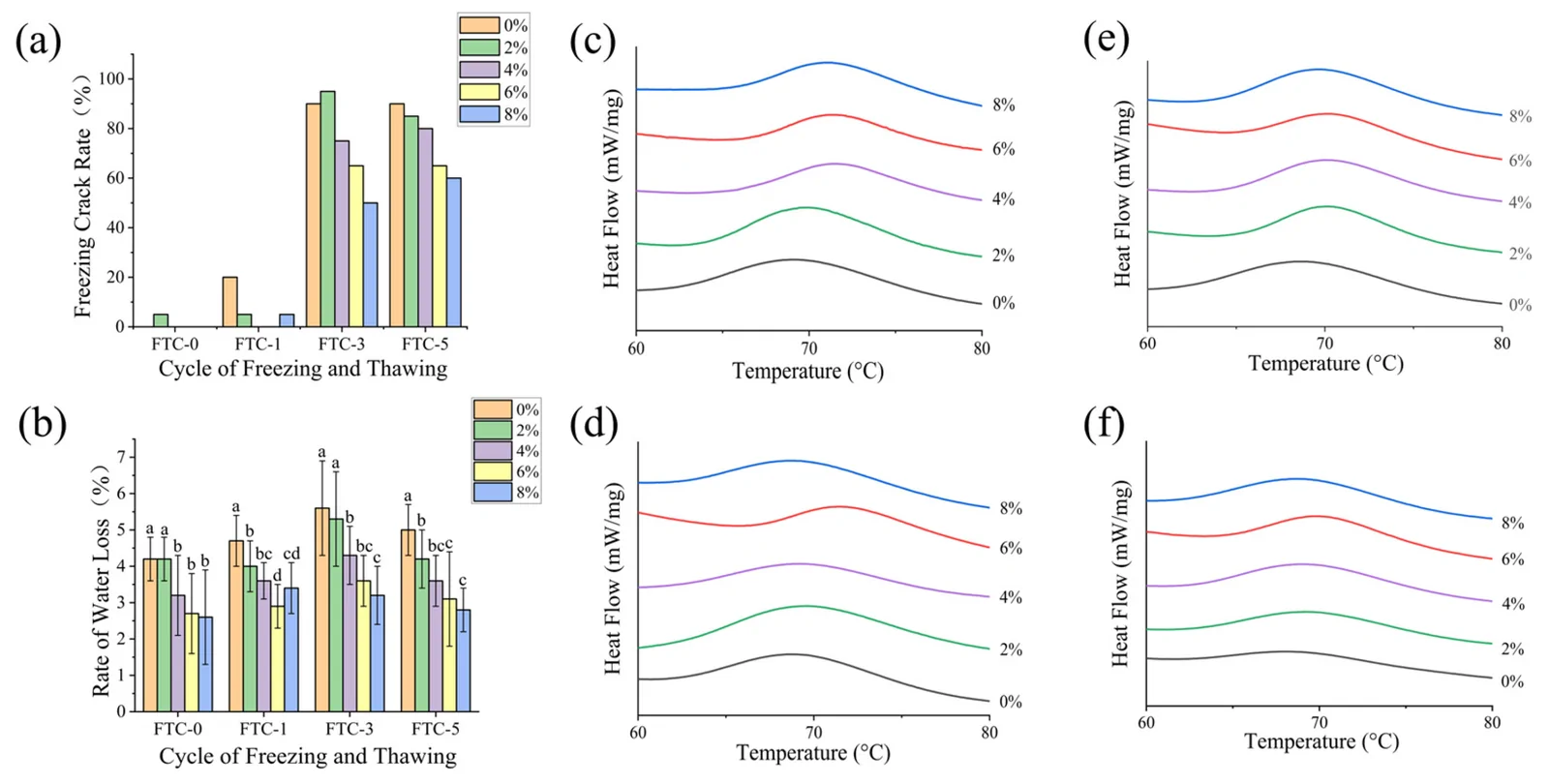
Effect of PGRS on the (**a**) cracking rate and (**b**) water loss rate of GRDs, as well as on the thermal properties of GRDs at (**c**) FTC-0, (**d**) FTC-1, (**e**) FTC-3, and (**f**) FTC-5. In panel (**b**), different lowercase letters indicate significant differences among PGRS substitution levels within the same freeze–thaw cycle condition (*p* < 0.05). Groups sharing at least one lowercase letter are not significantly different.

It has been reported that repeated freeze–thaw cycles significantly promote ice crystal growth and phase separation in GRD [28]. As shown in Figure S1 and Figure 2a, the control group exhibited relatively high cracking rates, whereas cracking rates generally decreased with increasing PGRS substitution levels. This reduction may be attributed to the ability of PGRS to increase the proportion of tightly bound water in GRDs, thereby accelerating the freezing process, reducing internal and external pressure, and minimizing cracking. Additionally, the amorphous structure of PGRS reduces the rigid arrangement of starch granules, providing GRDs with better extensibility and a lower risk of cracking during repeated freeze–thaw cycles [29]. As presented in Figure 2b, the incorporation of PGRS at substitution levels of 4–8% significantly (*p* < 0.05) reduced water loss in GRDs. This phenomenon may be explained by the enhanced water-binding interactions within the starch network facilitated by PGRS, effectively minimizing water exudation throughout repeated freeze–thaw cycles.

### 3.4. Comparative Effects of Native Rice Starch and PGRS

As shown in Table 1, native rice starch and PGRS produced different effects on the textural properties of GRDs after three freeze–thaw cycles. Compared with the 0% control, the 6% native rice starch group showed numerically lower hardness, but the difference was not significant. Chewiness and springiness also showed no significant differences between these two groups. In contrast, the 6% PGRS group exhibited significantly lower hardness and chewiness and significantly higher springiness than did the native rice starch group. These results indicate that native rice starch substitution alone did not reproduce the reductions in firmness and chewing resistance observed with PGRS. Compared with the 0% control, the PGRS group showed significantly lower hardness, whereas the differences in chewiness and springiness did not reach statistical significance.

Furthermore, the native rice starch group exhibited significantly higher cohesiveness and resilience than either the 0% control or the PGRS groups, while neither parameter differed significantly between the control and PGRS groups. This indicates that native rice starch itself affected the texture of GRDs, although its effects depended on the parameter examined. Taken together, these findings suggest that at a 6% substitution level after three freeze–thaw cycles, pregelatinization made the main contribution to the softer, less chewy texture observed with PGRS.

Under FTC-3 conditions, both the cracking rate and the water loss rate of the 6% native rice starch group were numerically higher than those of the 6% PGRS group (Table 2). This indicates that replacing part of the glutinous rice flour with native rice starch alone failed to reproduce the improvement in freeze–thaw stability achieved with PGRS. In contrast, pregelatinization further reduced the cracking rate and water loss rate of the dumplings, suggesting that the improvement in freeze–thaw stability was more closely associated with the pregelatinization process. These findings also corroborate the results of the comparative texture analysis, demonstrating that pregelatinization is an important factor in improving the quality of glutinous rice dumplings after freeze–thaw events.

### 3.5. Thermal Properties

DSC was employed to investigate the gelatinization behavior of glutinous rice flour by measuring its thermal characteristics, including the onset temperature (T_o_), peak temperature (T_p_), and end temperature (T_e_), as well as the gelatinization enthalpy (ΔH). DSC provides quantitative measurements of heat flow associated with gelatinization, in which the endothermic peaks are indicative of melting. The gelatinization temperature is characteristic of the starch type and depends on the glass transition of the amorphous fraction of the starch. As illustrated in Figure 2c–f, glutinous rice flour exhibited a single broad peak, which was attributed to the melting of starch crystallites and double-helices [30]. The ΔH is positively correlated with the degree of order in the long-range and short-range structures of starch. Specifically, a higher ΔH value indicates a more complete internal crystal structure and more regular arrangement of starch, requiring more energy to disrupt the crystal structure during gelatinization.

As depicted in Table 3, the incorporation of PGRS led to notable changes in the thermal parameters. With the increase in the addition of PGRS, the T_P_ initially increased and subsequently decreased. Under FTC-0 and FTC-1, ΔH decreased to a minimum at 6% PGRS and then increased at 8%, whereas under FTC-3 and FTC-5, ΔH continued to decrease up to 8% PGRS. This phenomenon can be attributed to the structural characteristics of PGRS itself: the pregelatinization process has partially disrupted the crystalline structure of the starch, resulting in reduced crystallinity and decreased ordered structure [31]. Under FTC-0 and FTC-1, the increase in ΔH at 8% PGRS relative to that at 6% PGRS suggests the formation of more organized structures. The underlying reason could be that excess PGRS permeated into the amorphous regions of glutinous rice starch granules along with water, together forming relatively stable complexes. These complexes may require more energy input to decompose during the gelatinization process, thereby leading to an increase in ΔH [12]. This nonlinear change indicates that PGRS plays a dual role in the starch system, acting both as a structural diluent and an interaction mediator. This characteristic also provides a key theoretical basis for optimizing the processing technology of glutinous rice products by regulating the PGRS addition amount in subsequent studies.

### 3.6. Dynamic Rheological Properties

**Figure 3:**
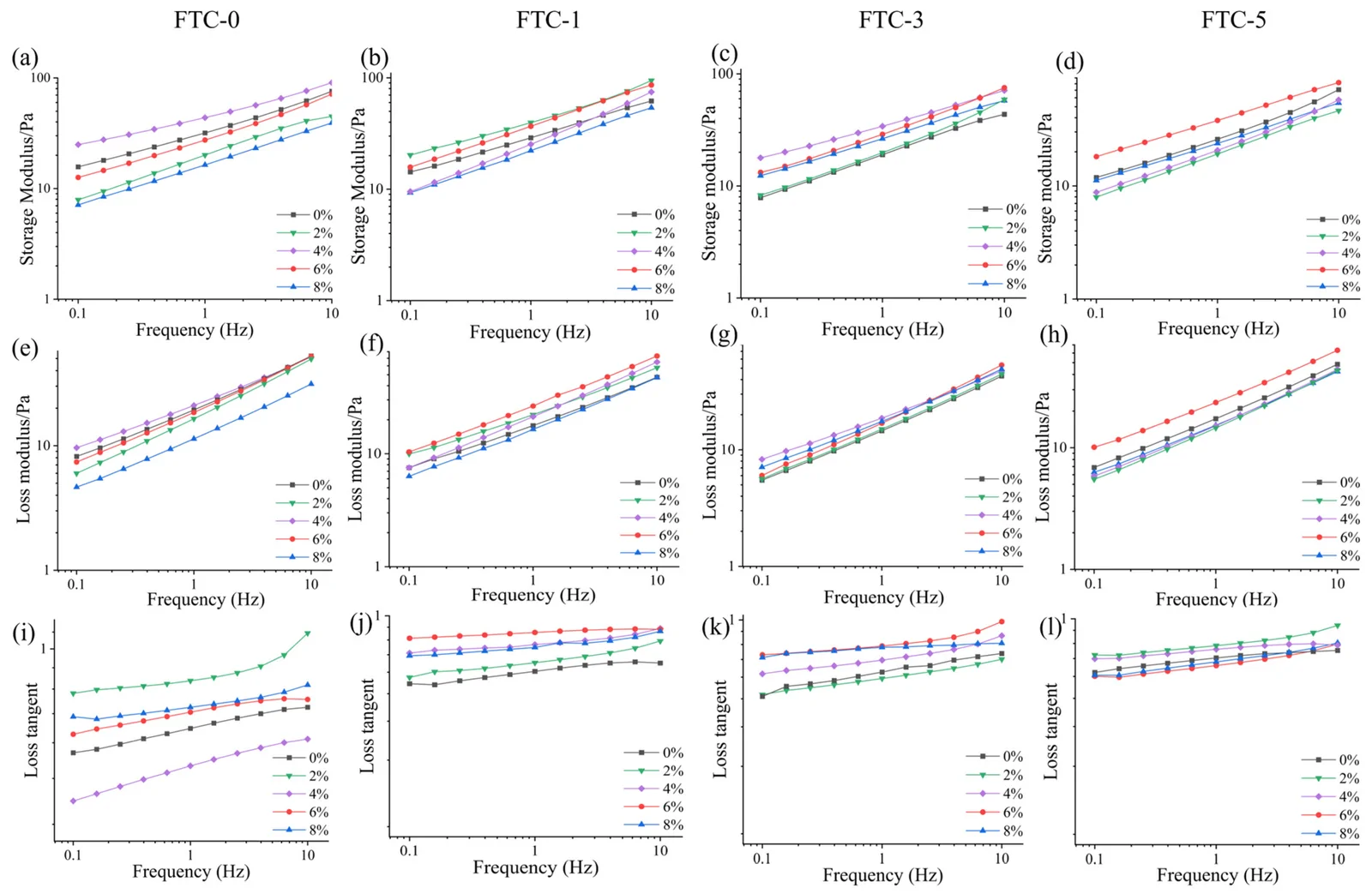
Effect of different frequency on GRD (**a**–**d**) storage modulus, (**e**–**h**) loss modulus, and (**i**–**l**) loss tangent under different FTCs. Each treatment was measured in triplicate (*n* = 3).

As a core technology for characterizing the structure of food colloid systems, dynamic rheological measurements can be used to characterize the interactions between the dispersed phase and the continuous phase. The core advantage of this technology lies in its ability to distinguish the contribution ratios of elasticity (the ability to store energy) and viscosity (the ability to dissipate energy) to the total stress by scanning the stress response under different frequency or temperature conditions in a non-destructive manner, thereby deducing the network structure state inside the system [32]. As shown in Figure 3, PGRS had a significant influence on the viscoelastic behavior of GRD gels, including the storage modulus (G′), loss modulus (G″), and loss tangent (tan δ) of GRDs under different freeze–thaw cycles. Among these parameters, G′ reflects the gel’s ability to store energy from elastic deformation and is directly related to the texture hardness and structural water-holding capacity of GRD; G″ represents the viscous characteristic of the gel that dissipates energy and is associated with the chewiness and extensibility of the product. Under FTC-3, the gels containing 4% and 6% PGRS generally exhibited higher storage modulus (G′) and loss modulus (G″) values than those of the control. This enhancement could be attributed to the fact that the hydroxyl groups on the surface of an appropriate number of PGRS molecules form hydrogen bond cross-linking with glutinous rice starch chains, promoting the construction of a highly ordered continuous phase network between starch molecules. This network can not only buffer the mechanical pressure generated by ice crystal growth during the freeze–thaw process but also reduce network collapse caused by water migration, thereby significantly enhancing the viscoelasticity and structural stability of the gel [33]. It is worth noting that this rheological property result is completely consistent with the texture analysis data in the document: the GRDs in the 4–6% PGRS group exhibit better hardness and chewiness, and the rate of texture deterioration after freeze–thaw events is significantly reduced, which further verifies the reliability of the dynamic rheological test conclusions. Furthermore, throughout the entire frequency sweep process, the tan δ values of all samples were generally less than 1 throughout the frequency sweep, which indicated that the GRD gel exhibits typical weak gel characteristics, meaning that the system possesses both elastic and viscous properties, with elasticity consistently dominating. In the low-frequency range, the lower tan δ values suggested that the starch gels exhibit predominantly elastic solid characteristics. As the frequency increased, the tan δ values rose, reflecting an increase in viscous fluid characteristics.

### 3.7. FT-IR Analysis

**Figure 4:**
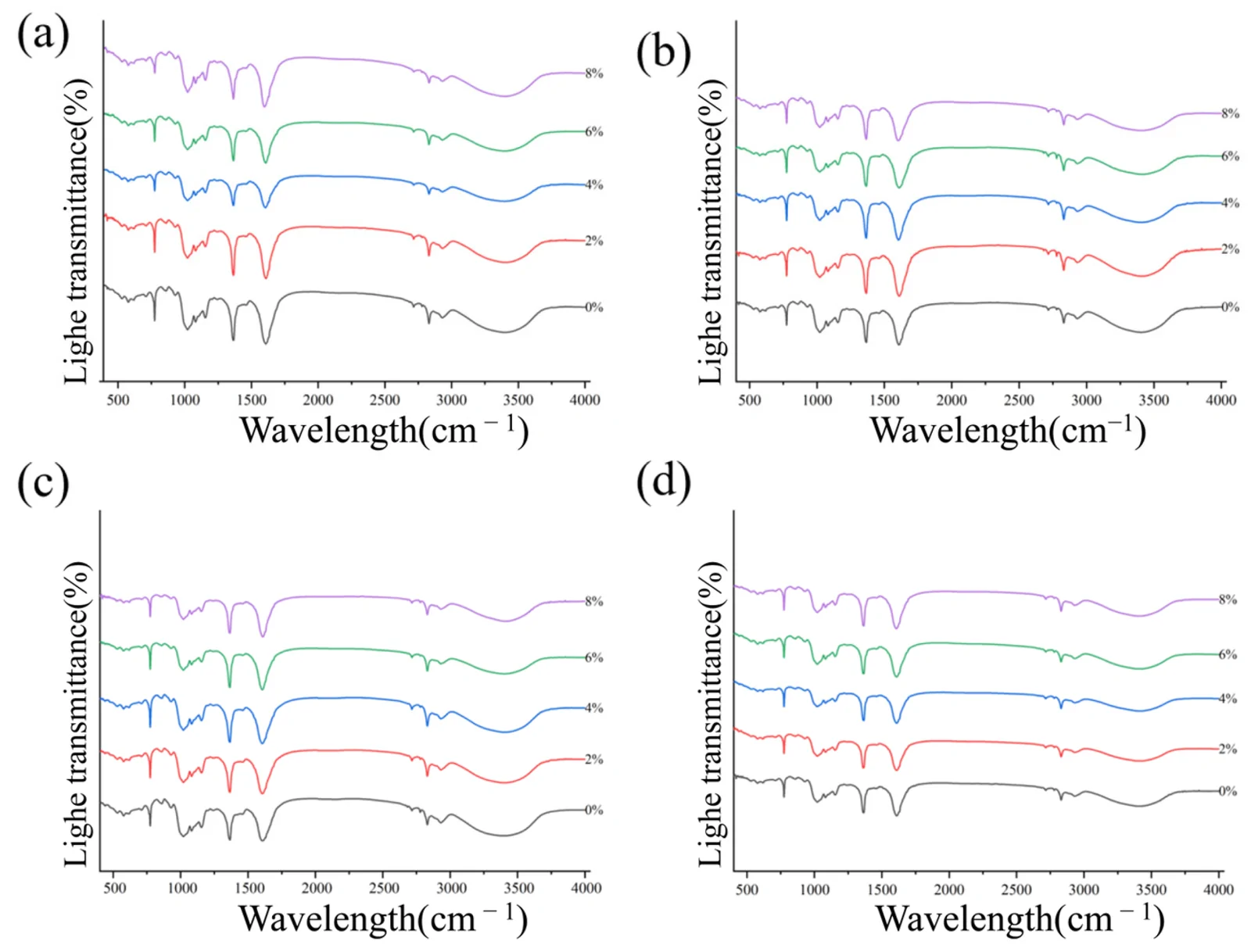
FT-IR spectra of GRDs with different PGRS substitution levels after (**a**) FTC-0, (**b**) FTC-1, (**c**) FTC-3, and (**d**) FTC-5.

FT-IR exhibits high sensitivity to changes in the molecular-level structure of starch, and it can be employed to determine the presence and strength of intermolecular hydrogen bonds in the sample. As can be seen from the Figure 4, no new absorption peaks appear after the addition of PGRS, which indicates that the interaction between PGRS and glutinous rice starch occurs primarily through hydrogen bonds rather than covalent bonding.

The FT-IR spectra were responsive to the oscillation frequency of the chemical bonds, and they could be used to measure the short-range structure of starch. The ratio of absorbance at 1047 cm^−1^ to that at 1022 cm^−1^ (R_1047_/_1022_) was used to quantify the degree of order in starch samples [34].

Table 4 presents the statistical analysis of R_1047/1022_ values for samples subjected to various FTCs. The results show that under the same FTC, as the amount of PGRS increases, the R_1047/1022_ value gradually decreases, reaching its minimum at 6%, but slightly rebounding at 8%. This indicates that the ordered structure first decreases and then recovers, confirming the reliability of the thermal properties analysis. The reduction in ordered structure is primarily attributed to the partial disruption of the crystalline structure of starch by pregelatinization, resulting in decreased crystallinity and reduced ordered structure. Conversely, at a substitution rate of 8%, the increase in ordered structure may be due to excessive exposure of hydroxyl groups promoted by pregelatinization, which enhances hydrogen bonding between adjacent double helices and free starch chains, thereby facilitating the formation of helical structures and improving the degree of order [35].

### 3.8. Analysis of the Water Status via LF-NMR

**Figure 5:**
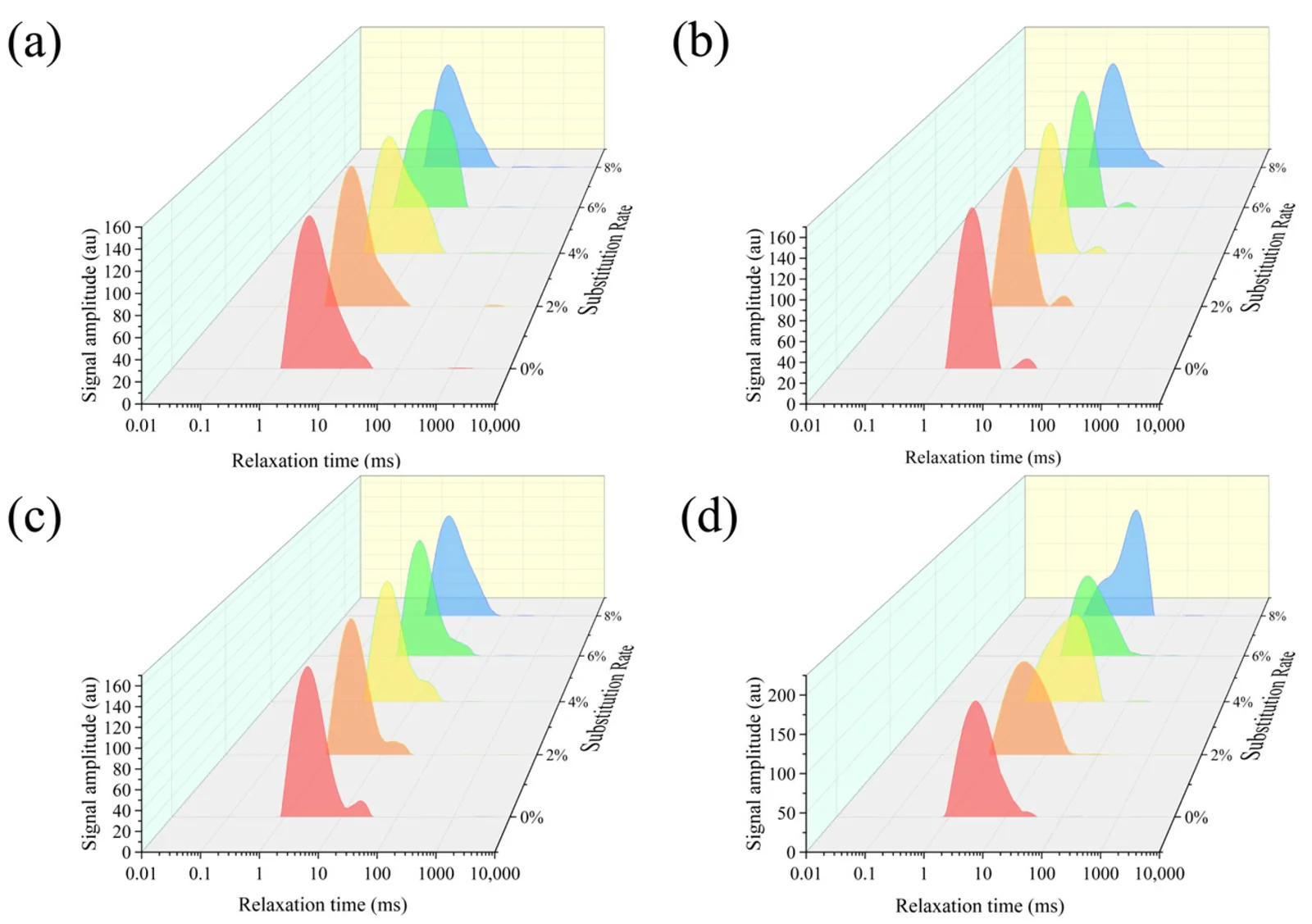
Effect of PGRS on the LF-NMR of GRDs after (**a**) FTC-0, (**b**) FTC-1, (**c**) FTC-3, and (**d**) FTC-5. The red, orange, yellow, green, and blue curves represent PGRS substitution levels of 0%, 2%, 4%, 6%, and 8%, respectively.

The content and distribution of water in GRDs critically influence its overall quality. LF-NMR is a rapid non-destructive detection technique used to analyze the distribution of water in food systems. The total area of all peaks in the relaxation spectrum (Figure 5) exhibits a direct and quantitative correlation with the sample’s total water content. Within GRDs, water exists primarily in three states: tightly bound water T_21_ (<10 ms), weakly bound water T_22_ (10–1000 ms), and free water T_23_ (>1000 ms). Tightly bound water is primarily immobilized within starch granules, weakly bound water is adsorbed on the surface of the granules, and free water remains mobile and unbound throughout the system. The proportions of the peak areas corresponding to these characteristic states are quantitatively represented by the parameters A_21_, A_22_, and A_23_, respectively.

As evidenced in Table S1, when the substitution ratio of PGRS was 4% and 6%, the total water retention in the freeze–thawed GRD was markedly enhanced. This improvement could be attributed to the water-retaining film formed by PGRS in GRDs, which effectively hinders the migration of internal water to the surface, along with subsequent evaporation [36], thereby reducing water loss during gelatinization and freeze–thaw processes. Meanwhile, the proportion of tightly bound water increased significantly, while the proportions of weakly bound water and free water decreased significantly, indicating a reduction in the mobility of internal water and the freedom of hydrogen protons. The enhanced water binding may result from the abundant exposed hydroxyl groups on the surface of PGRS molecules, which exhibit strong hydrophilic affinity and promote the conversion of weakly bound water and free water into tightly bound water [37]. Furthermore, studies have shown that PGRS can form a three-dimensional gel network structure in the glutinous rice starch dough, further limiting the movement of water molecules [12]. These mechanisms collectively account for the observed decrease in freeze cracking and water loss rates as the substitution ratio increases.

### 3.9. Scanning Electron Microscopy (SEM)

**Figure 6:**
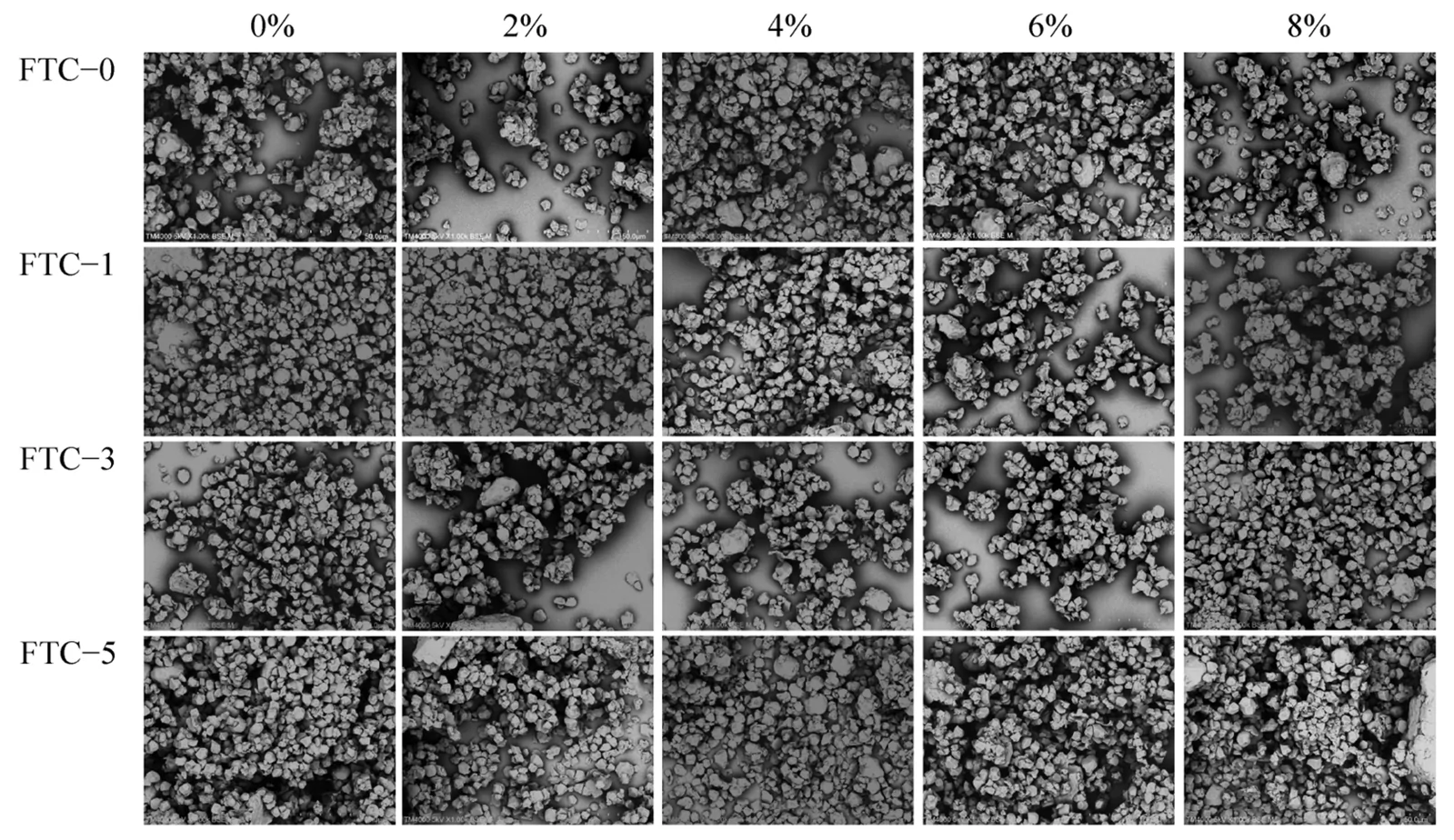
Effect of PGRS on the microstructure of GRDs after FTC-0, FTC-1, FTC-3, and FTC-5 (1000× magnification).

As can be seen in Figure 6, with the increase in PGRS substitution rate, SEM observations revealed significant improvements in the microstructure of freeze-dried GRD flour subjected to multiple freeze–thaw cycles. Specifically, when the PGRS substitution rate ranged between 2% and 6%, the samples exhibited higher structural integrity, relatively smooth particle surfaces, and fewer, yet more uniformly distributed, pores. This may be attributed to the strong gel-forming properties of PGRS, which reinforces the structural resistance of GRDs against ice-crystal-induced damage during freeze–thaw cycles. Notably, although the 8% substitution group still maintained relatively good structural integrity, to some extent, some samples showed particle aggregation on the surface, along with a reduction in pore number and uneven pore distribution, suggesting that excessive PGRS substitution rates may diminish the improvement effects [38]. In contrast, the control group samples without PGRS displayed significant surface collapse, cracks, and voids after undergoing freeze–thaw cycles, indicating that repeated freezing and thawing of ice crystals damaged the starch granule structure. Especially under multiple freeze–thaw cycles (FTC-3, FTC-5), the microstructure of the control group samples became loose, which could lead to an increase in water migration paths, thereby accelerating the quality deterioration of the GRDs. Overall, a moderate addition of PGRS (2–6%) helps improve the microstructural stability of GRDs under freeze–thaw cycles, possibly by providing a more uniform network structure and stronger water retention capability, ultimately improving freeze–thaw resistance of GRDs.

## 4. Conclusions

Focusing on the core issue of quality deterioration in GRDs caused by repeated freeze–thaw cycles during storage and transportation, this study systematically investigated the regulatory effects of PGRS at different addition ratios (0%, 2%, 4%, 6%, and 8%) on the quality and freeze–thaw stability of GRDs. The results demonstrated that the addition of PGRS mitigated the quality deterioration caused by repeated freezing and thawing and significantly enhanced its water holding capacity and freeze–thaw stability. Key findings revealed that PGRS reduced the ordered structure of the gel matrix and increased the content of bound water, thereby improving microstructural stability and reducing ΔH. These changes contributed to greater textural stability and reduced cracking and water loss in PGRS-incorporated GRDs. The native rice starch comparison suggests that pregelatinization played a key role in reducing hardness and chewiness and improving the freeze–thaw stability of GRDs at 6% substitution after three freeze–thaw cycles. Taken together, the water distribution and structural results support a mechanism in which PGRS enhances water binding and modifies the starch network, contributing to improved freeze–thaw stability. Furthermore, rheological and morphological analyses confirmed that moderate PGRS levels promoted a continuous and uniform network within the starch gel, leading to improved viscoelastic properties and reduced structural collapse. The results revealed different responses to PGRS substitution across quality parameters. Moderate substitution was more favorable for textural properties and microstructural uniformity, whereas higher substitution levels benefited certain parameters, as reflected by the highest soup transmittance at 8% PGRS under FTC-1. However, the partial aggregation and less uniform pore distribution observed at 8% suggested that excessive substitution may compromise structural uniformity. Taken together, the results confirmed that 6% PGRS provided the most favorable overall balance between textural properties, soup clarity after repeated freeze–thaw cycles, and microstructural uniformity under the conditions investigated. In conclusion, PGRS serves as an effective functional ingredient for enhancing the freeze–thaw stability and sensory quality of GRDs. These findings provide both theoretical and practical insights for the industrial production of quick-frozen rice-based products, supporting the application of PGRS as a natural and efficient modifier to extend shelf life and maintain product quality under fluctuating frozen storage conditions.

## Figures and Tables

**Table 1:** Textural properties of GRD in different treatment groups after three freeze–thaw cycles (FTC-3).

Parameter	0% Control (*n* = 3)	6% Native Rice Starch (*n* = 6)	6% PGRS (*n* = 3)
Hardness (g)	845.48 ± 70.20 ^a^	768.52 ± 42.11 ^a^	512.54 ± 14.40 ^b^
Chewiness (g)	510.44 ± 49.26 ^ab^	513.75 ± 27.43 ^a^	355.40 ± 8.32 ^b^
Springiness	0.8403 ± 0.0576 ^ab^	0.8227 ± 0.0290 ^b^	0.9280 ± 0.0034 ^a^
Cohesiveness	0.7193 ± 0.0168 ^b^	0.8132 ± 0.0172 ^a^	0.7473 ± 0.0035 ^b^
Resilience	0.3798 ± 0.0086 ^b^	0.4768 ± 0.0118 ^a^	0.3888 ± 0.0043 ^b^

Note: Values are presented as means ± SD; sample sizes are indicated in the column headings. Different lowercase letters within the same row indicate significant differences among treatments (*p* < 0.05). Groups sharing at least one letter are not significantly different. Comparisons were performed using Welch’s one-way ANOVA followed by Games–Howell multiple comparisons.

**Table 2:** Cracking rate and water loss rate of GRDs in different treatment groups after three freeze–thaw cycles (FTC-3).

Treatment Group	Cracking Rate (%)	Water Loss Rate (%)
Control (0%)	90	5.6
6% PGRS group	65	3.6
6% Native Rice Starch group	85	4.33 ± 2.10

**Table 3:** Thermal properties of GRDs under different FTCs.

Freeze–thaw Cycles	Substitution Ratio	T_o_ (°C)	T_p_ (°C)	T_e_ (°C)	ΔH (J/g)
FTC-0	0%	63.69 ± 0.47 ^e^	69.10 ± 0.14 ^d^	76.16 ± 0.30 ^a^	1.48 ± 0.36 ^a^
	2%	64.53 ± 0.42 ^c^	69.89 ± 0.23 ^c^	75.87 ± 0.64 ^ab^	1.37 ± 0.33 ^b^
	4%	66.12 ± 0.40 ^b^	71.71 ± 0.42 ^a^	75.40 ± 0.46 ^bc^	1.25 ± 0.13 ^c^
	6%	66.36 ± 0.84 ^a^	71.75 ± 0.13 ^a^	74.28 ± 0.15 ^d^	1.03 ± 0.30 ^e^
	8%	65.91 ± 0.35 ^d^	70.84 ± 0.84 ^b^	74.59 ± 0.64 ^cd^	1.14 ± 0.44 ^d^
FTC-1	0%	61.97 ± 0.28 ^d^	68.67 ± 0.78 ^c^	76.78 ± 0.59 ^a^	1.47 ± 0.11 ^a^
	2%	63.45 ± 0.30 ^b^	69.42 ± 0.85 ^b^	75.64 ± 0.26 ^ab^	1.33 ± 0.51 ^b^
	4%	63.68 ± 0.21 ^b^	69.47 ± 0.30 ^b^	75.37 ± 0.73 ^b^	1.28 ± 0.38 ^b^
	6%	67.23 ± 0.16 ^a^	71.68 ± 0.27 ^a^	75.41 ± 0.61 ^b^	1.11 ± 0.12 ^c^
	8%	62.89 ± 0.57 ^c^	68.91 ± 0.14 ^c^	73.39 ± 0.25 ^c^	1.21 ± 0.82 ^bc^
FTC-3	0%	63.44 ± 0.40 ^b^	69.19 ± 0.12 ^c^	76.56 ± 0.59 ^a^	1.43 ± 0.57 ^a^
	2%	65.03 ± 0.16 ^a^	70.26 ± 0.21 ^b^	75.85 ± 0.23 ^ab^	1.24 ± 0.78 ^b^
	4%	65.46 ± 0.11 ^a^	70.32 ± 0.23 ^b^	75.77 ± 0.25 ^ab^	1.18 ± 0.50 ^bc^
	6%	65.58 ± 0.18 ^a^	70.83 ± 0.22 ^a^	75.25 ± 0.44 ^b^	1.16 ± 0.42 ^bc^
	8%	64.92 ± 0.90 ^a^	70.04 ± 0.62 ^c^	75.16 ± 0.25 ^b^	1.05 ± 0.71 ^c^
FTC-5	0%	62.97 ± 0.92 ^e^	68.48 ± 0.71 ^d^	77.51 ± 0.30 ^a^	1.36 ± 0.35 ^a^
	2%	64.01 ± 0.50 ^c^	69.53 ± 0.11 ^ab^	75.71 ± 0.64 ^b^	1.21 ± 0.64 ^b^
	4%	64.28 ± 0.14 ^b^	69.30 ± 0.71 ^b^	75.24 ± 0.46 ^b^	1.15 ± 0.11 ^bc^
	6%	64.84 ± 0.50 ^a^	69.76 ± 0.13 ^a^	75.14 ± 0.15 ^b^	1.12 ± 0.04 ^c^
	8%	63.68 ± 0.16 ^d^	68.85 ± 0.78 ^c^	75.20 ± 0.64 ^b^	1.09 ± 0.13 ^c^

Note: The means ± SD with different lowercase letters in the same column indicate significant differences between them (*p* < 0.05).

**Table 4:** The R_1047/1022_ of GRDs under different FTCs.

Substitution Ratio	FTC-0	FTC-1	FTC-3	FTC-5
0%	1.1374 ± 0.0104 ^a^	1.1723 ± 0.0189 ^a^	1.2304 ± 0.0102 ^a^	1.3207 ± 0.0180 ^a^
2%	1.1041 ± 0.0185 ^b^	1.1433 ± 0.0046 ^ab^	1.1164 ± 0.0181 ^cd^	1.2003 ± 0.0066 ^b^
4%	1.1040 ± 0.0097 ^b^	1.1062 ± 0.0134 ^cd^	1.1296 ± 0.0184 ^c^	1.1316 ± 0.0078 ^c^
6%	1.0537 ± 0.0191 ^c^	1.0890 ± 0.0187 ^d^	1.0947 ± 0.0181 ^d^	1.1139 ± 0.0165 ^c^
8%	1.1041 ± 0.0104 ^b^	1.1302 ± 0.0196 ^bc^	1.1986 ± 0.0077 ^b^	1.1994 ± 0.0184 ^b^

Note: The means ± SD, with different lowercase letters in the same column indicating significant differences between them (*p* < 0.05).

## Data Availability

The original contributions presented in this study are included in the article/Supplementary Materials. Further inquiries can be directed to the corresponding author.

## Supplementary Materials

The following supporting information can be downloaded at: https://www.mdpi.com/article/10.3390/foods15213929/s1, Figure S1: GRD of (a) FTC-0, (b) FTC-1, (c) FTC-3 and (d) FTC-5; Table S1: Effect of PGRS on water distribution of GRDs.

## References

[1] Li Y., Ding G., Yokoyama W., Zhong F. (2018). Characteristics of annealed glutinous rice flour and its formation of fast-frozen dumplings. J. Cereal Sci..

[2] Shi C., Zhu S., Ding G., Du C., Huang D., Li Y. (2021). Modulating Structure and Properties of Glutinous Rice Flour and Its Dumpling Products by Annealing. Processes.

[3] Qi W., Zhang G., Mei J., Zhang C., Xie J. (2023). Optimization of freezing methods and composition of frozen rice dough reconstituted by glutinous rice starch and gluten. Int. J. Biol. Macromol..

[4] Chen L., Tan H., Feng R., Ma L., Zhang Y., Yi H., Yin L., Liu W., Hu L., Zhu W. (2023). Effect of modified starches on the quality of skins of glutinous rice dumplings. Int. J. Biol. Macromol..

[5] Huang T., Ouyang D., Sang S., Li C., Wang X., Wang X., Xing J., Luo X. (2024). Effect of Water Content in Semidry Grinding on the Quality of Glutinous Rice Flour. Foods.

[6] Li D., Wan Y., Zhang J., Zhang Y., He R., Sang S., Xing J., Luo X. (2025). Effect of electron beam irradiation on edible quality maintenance of high moisture rice grains. J. Cereal Sci..

[7] Jin Z., Ren Y., Wan Y., Ouyang D., Zhang H., Huang K., Sang S., Liu Y., Xing J., Luo X. (2026). Effect of dielectric barrier discharge cold plasma treatment on high moisture brown rice and its starch characteristics. Food Bioprocess Technol..

[8] Chang T., Bian L., Zhang X., Chen S., Lyu Y., Li G., Zhang C. (2024). Impacts of transglutaminase on the processing and digestion characteristics of glutinous rice flour: Insight of the interactions between enzymic crossing-linked protein and starch. Food Res. Int..

[9] Huang F., Hu H., Ouyang K., Zhang Q., Li G., Zhong J., Yu L., Zhao Q. (2025). The processing quality characteristics, freeze-thaw stability, and retrogradation properties of rice cakes affected by four hydrocolloids: Xanthan gum, guar gum, carrageenan, and konjac gum. Int. J. Biol. Macromol..

[10] Sugiura F., Ito S., Arai E. (2017). Effect of pregelatinized starch paste on the ease of swallowing high-moisture content bread. J. Food Eng..

[11] Sun X., Li W., Hu Y., Zhou X., Ji M., Yu D., Fujita K., Tatsumi E., Luan G. (2018). Comparison of pregelatinization methods on physicochemical, functional and structural properties of tartary buckwheat flour and noodle quality. J. Cereal Sci..

[12] Wang H., Xiao N., Wang X., Zhao X., Zhang H. (2019). Effect of pregelatinized starch on the characteristics, microstructures, and quality attributes of glutinous rice flour and dumplings. Food Chem..

[13] Li Y., Li C., Ban X., Cheng L., Hong Y., Gu Z., Li Z. (2021). New insights into the alleviating role of starch derivatives on dough quality deterioration caused by freeze. Food Chem..

[14] Arocas A., Sanz T., Fiszman S.M. (2009). Clean label starches as thickeners in white sauces. Shearing, heating and freeze/thaw stability. Food Hydrocoll..

[15] Li J., Berger F. (2012). Endosperm: Food for humankind and fodder for scientific discoveries. New Phytol..

[16] Wang L., Xie B., Xiong G., Wu W., Wang J., Qiao Y., Liao L. (2013). The effect of freeze–thaw cycles on microstructure and physicochemical properties of four starch gels. Food Hydrocoll..

[17] Zhu Y., Dong C., Chi F., Gu X., Liu L., Yang L. (2024). Effects of Cactus Polysaccharide on Pasting, Rheology, Structural Properties, In Vitro Digestibility, and Freeze-Thaw Stability of Rice Starch. Foods.

[18] Ding X., Wang L., Li T., Wang F., Quan Z., Zhou M., Huo Z., Qian J. (2021). Pre-Gelatinisation of Rice Flour and Its Effect on the Properties of Gluten Free Rice Bread and Its Batter. Foods.

[19] Yu R., Deng Y., Wan Y., Zhan X., Dai D., Yan F., Dai Z., Ouyang D., Xing J., Luo X. (2026). Heat-modulated brown rice starch-protein: Physicochemical, functional, and digestibility properties. J. Food Eng..

[20] Yulianingsih R., Gohtani S. (2019). Dispersion characteristics of pregelatinized waxy rice starch and its performance as an emulsifier for oil-in-water emulsions: Effect of gelatinization temperature and starch concentration. Food Hydrocoll..

[21] Guan H., Li Y., Qin X., Chen Z., Wang H., Zeng Z., Liu X. (2024). Effect of sodium alginate on freeze-thaw stability of deacetylated konjac glucomannan gel. J. Food Eng..

[22] Zhai Y., Xing J., Luo X., Zhang H., Yang K., Shao X., Chen K., Li Y. (2021). Effects of Pectin on the Physicochemical Properties and Freeze-Thaw Stability of Waxy Rice Starch. Foods.

[23] Li D., Li H., Tao Y., Li G., Xu E., Han Y., Ding Y. (2023). Static magnetic field improvement of the quality of rice dumpling subjected to freeze–thaw cycles: Roles of phase transition of water and changes in structural and physicochemical properties of glutinous rice flour. Food Res. Int..

[24] Xu K., She Z., Wang H., Liu X., Zhang Y., Chi C., Zhang H. (2021). Textural quality of sweet dumpling: Effect of germination of waxy rice. Int. J. Food Sci. Technol..

[25] Nakorn K.N., Tongdang T., Sirivongpaisal P. (2009). Crystallinity and Rheological Properties of Pregelatinized Rice Starches Differing in Amylose Content. Starch.

[26] Kälviäinen N., Roininen K., Tuorila H. (2000). Sensory characterization of texture and flavor of high viscosity gels made with different thickeners. J. Texture Stud..

[27] Morawicki R.O., Siebenmorgen T., Cho M.J. (2009). Study of solids leached during rice cooking using the transmittance at 650 nm. J. Food Qual..

[28] Tao X., Zhang Y., Chen F., Huang Y., Chen P. (2021). Effects of sucrose on pasting, thermal, rheological and textural properties of native and alcohol-alkali-treated waxy rice starch. Int. J. Biol. Macromol..

[29] Chieregato Maniglia B., Carregari Polachini T., Norwood E.-A., Le-Bail P., Le-Bail A. (2021). Thermal technologies to enhance starch performance and starchy products. Curr. Opin. Food Sci..

[30] Qiao D., Yu L., Liu H., Zou W., Xie F., Simon G., Petinakis E., Shen Z., Chen L. (2016). Insights into the hierarchical structure and digestion rate of alkali-modulated starches with different amylose contents. Carbohydr. Polym..

[31] Santos R.d., Franco C.M.L., Mischan M.M., Leonel M. (2019). Behavior of Sweet Potato Starch After Spray-Drying Under Different Pretreatment Conditions. Starch.

[32] Zhang X., Lin X., Xu B. (2024). Morphological, physicochemical, and pasting properties of pre-gelatinized starch prepared by high-pressure homogenizer: A comparative study on starches from different resources. Food Res. Int..

[33] Yang X., Liu L., Mahmood N., Qin J., Zhao Q., Song X. (2025). Response of texture, rheological characteristics and starch molecular structure of super hybrid indica rice to different nitrogen fertilizer levels. Carbohydr. Polym..

[34] Gao J., Zhu L., Huang J., Li L., Yang Y., Xu Y., Wang Y., Wang L. (2021). Effect of dandelion root polysaccharide on the pasting, gelatinization, rheology, structural properties and in vitro digestibility of corn starch. Food Funct..

[35] Wang S., Li C., Copeland L., Niu Q., Wang S. (2015). Starch Retrogradation: A Comprehensive Review. Compr. Rev. Food Sci. Food Saf..

[36] Fu Z., Wang L., Zou H., Li D., Adhikari B. (2014). Studies on the starch-water interactions between partially gelatinized corn starch and water during gelatinization. Carbohydr. Polym..

[37] Thongtan R.R., Sriroth K.R. (2011). Effect of Cassava Starch Destructuration to Mechanical Properties of Compostable Blends. Adv. Mater. Res..

[38] Hu J.-P., Wang S.-Y., Wang D.-Q., Zong K.-L., Yang J.-T. (2022). Effects of carrot powder on properties of pre-gelatinized waxy rice starch. Food Sci. Technol..

