# Supplementary material for: Effect of Pregelatinized Rice Starch on the Quality and Freeze–Thaw Stability of Glutinous Rice Dumplings
Source: Foods. 2026 Nov 2;15(21):3929. doi: 10.3390/foods15213929 (PMC13762813; doi:10.3390/foods15213929)
Supplement: Supplementary file 1 [file foods-15-03929-s001.zip › foods-4628519-supplementary.pdf]

Supplementary Material

Effect of Pregelatinized Rice Starch on the Quality and Freeze-Thaw Stability of Glutinous Rice Dumpling

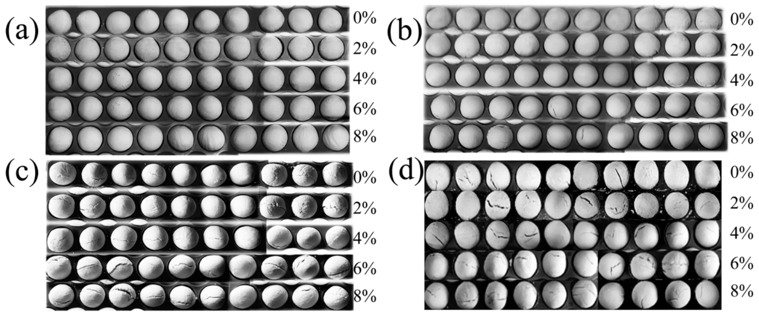

Figure S1. GRD of (a) FTC-0, (b) FTC-1, (c) FTC-3 and (d) FTC-5.

Table S1. Effect of PGRS on water distribution of GRDs

Freeze-thaw Cycle	Substitution Rate	A_{21}(%)	A_{22}(%)	A_{23}(%)	Total Peak Area
FTC-0	0%	98.55 ± 0.12^{c}	1.25 ± 0.12^{a}	0.20 ± 0.28^{a}	3855.83 ± 97.32^{c}
	2%	98.24 ± 0.15^{d}	1.30 ± 0.22^{a}	0.46 ± 0.41^{a}	3782.47 ± 125.04^{c}
	4%	99.49 ± 0.15^{b}	0.30 ± 0.02^{b}	0.20 ± 0.18^{a}	4219.62 ± 133.55^{b}
	6%	99.8 ± 0.02^{a}	0.20 ± 0.02^{b}	0.03 ± 0.12^{a}	4606.12 ± 136.42^{a}
	8%	98.64 ± 0.13^{c}	1.20 ± 0.28^{a}	0.14 ± 0.2^{a}	3961.06 ± 91.27^{c}
FCT-1	0%	97.13 ± 0.09^{c}	2.85 ± 0.10^{b}	0.02 ± 0.01^{b}	3101.33 ± 39.07^{d}
	2%	96.46 ± 0.04^{d}	3.50 ± 0.15^{a}	0.06 ± 0.05^{ab}	3342.41 ± 28.54^{c}
	4%	97.56 ± 0.09^{b}	2.36 ± 0.09^{c}	0.08 ± 0.03^{b}	3209.64 ± 56.62^{b}
	6%	97.87 ± 0.09^{a}	2.04 ± 0.17^{d}	0.09 ± 0.03^{b}	3287.41 ± 50.68^{ab}
	8%	97.91 ± 0.11^{a}	2.02 ± 0.16^{d}	0.07 ± 0.01^{ab}	3809.60 ± 44.22^{a}
FTC-3	0%	95.65 ± 0.24^{a}	4.30 ± 0.23^{a}	0.05 ± 0.02^{a}	3440.43 ± 35.01^{d}
	2%	96.83 ± 1.28^{ab}	3.11 ± 1.31^{ab}	0.06 ± 0.05^{a}	3508.76 ± 52.53^{d}
	4%	97.50 ± 0.16^{bc}	2.44 ± 0.20^{bc}	0.06 ± 0.05^{a}	3588.89 ± 30.87^{c}
	6%	98.08 ± 0.18^{bc}	1.77 ± 0.19^{bc}	0.16 ± 0.12^{a}	3709.98 ± 29.19^{b}
	8%	98.97 ± 1.42^{c}	1.01 ± 1.38^{c}	0.1 ± 0.03^{a}	4029.21 ± 58.35^{a}
FTC-5	0%	98.58 ± 0.38^{b}	1.37 ± 0.38^{a}	0.05 ± 0.02^{a}	3838.08 ± 57.58^{e}
	2%	99.06 ± 1.06^{ab}	1.42 ± 1.06^{a}	0.15 ± 0.20^{a}	4138.46 ± 66.37^{d}
	4%	99.74 ± 0.17^{a}	0.46 ± 0.18^{b}	0.02 ± 0.01^{a}	4376.93 ± 59.03^{c}
	6%	99.82 ± 0.05^{a}	0.31 ± 0.24^{b}	0.03 ± 0.03^{a}	4745.63 ± 28.75^{b}
	8%	99.76 ± 0.11^{a}	0.29 ± 0.08^{b}	0.05 ± 0.03^{a}	5280.91 ± 46.57^{a}

Note: The means ± SD with different lowercase letters in the same FTC group indicate significant differences ($p < 0.05$).
